# Identification of N7-methylguanosine related subtypes and construction of prognostic model in gastric cancer

**DOI:** 10.3389/fimmu.2022.984149

**Published:** 2022-10-10

**Authors:** Xiaoxiao Li, Hao Dong, Ling Chen, Yujie Wang, Zhibin Hao, Yingyi Zhang, Yuan Jiao, Zhiyue Zhao, Xiaobo Peng, Xianbao Zhan

**Affiliations:** ^1^ Department of Oncology, Changhai Hospital, Naval Military Medical University, Shanghai, China; ^2^ Department of Gastrointestinal Nutrition and Hernia Surgery, The Second Hospital of Jilin University, Changchun, China

**Keywords:** gastric cancer, N7-methylguanosine, molecule subtypes, prognosis, tumor microenvironment, immunotherapy, immune infiltration

## Abstract

**Background:**

N^7^-methylguanosine (m7G), one of the most common post-transcriptional modifications, can be present in tRNA, mRNA, and miRNA to mediate the progression of various tumors. However, the possible role of m7G in gastric cancer (GC) is still unknown.

**Materials and Methods:**

In this study, SNVs (single nucleotide variations), CNVs (copy number variations), and methylation of m7G-related genes (m7GRGs) were analyzed. The relationship between them and the expression of m7GRGs and prognosis of GC patients was explored. Based on 13 prognostic-related m7GRGs, 567 GC samples were classified into three subtypes using the ConsensusClusterPlus package. we compared survival status, clinical traits, immune cell infiltration, immune checkpoints, tumor microenvironment (TME), tumor immune dysfunction and exclusion (TIDE), and potential biological pathways among the three subtypes. Then, patients were again grouped into different genetic subtypes based on the DEGs among the three subtypes. In addition, a prognostic m7GRG_Score was constructed using five risk genes applicable to patients of any age, gender and stage. We also assessed tumor mutational burden (TMB), microsatellite instability (MSI), cancer stem cell (CSC) index, sensitivity of antineoplastic drugs, efficacy of anti-PD-1 and anti-CTLA4 immunotherapy between high and low m7GRG_Score groups. Finally, we established a nomogram based on m7GRG_Score and tumor stage to enhance the clinical application of the model. miRNAs and lncRNAs that could regulate expression of risk genes were searched.

**Results:**

SNVs, CNVs, and methylation of m7GRGs were associated with m7GRGs expression. However, they did not significantly affect the survival of GC patients. Our results also confirmed that patients in subtypes B and C and low m7GRG_Score groups had longer survival time, better clinical stage, more immune cell infiltration, fewer immune escape and dysfunction compared to subtype A and high m7GRG_Score groups. A low m7GRG_score was featured with increased microsatellite instability-high (MSI-H), TMB, and efficacy of immunotherapy.

**Conclusion:**

The m7GRG_Score model may become a beneficial tool for predicting prognosis and guiding personalized treatment in GC patients. These findings will improve our knowledge of m7G in GC and provide new methods for more effective treatment strategies.

## Introduction

As a major public health problem with high morbidity and mortality worldwide, tumor is one of the major diseases endangering human life. Gastric cancer (GC) is a strongly aggressive disease with a high degree of molecular and phenotypic heterogeneity (1). Although the morbidity and mortality of GC are declining in some developed countries, GC remains a common and fatal disease globally, especially in Northeast Asia and South America (2). In 2020, there were more than 1 million cases of gastric cancer worldwide, resulting in more than 768000 deaths. This makes gastric cancer the fifth most common cancer and the third leading cause of cancer-related death in the world (3, 4). Since patients with early gastric cancer usually have no symptoms, the early diagnosis rate of gastric cancer is very low. Most patients (>70%) are diagnosed at an advanced stage, when patients often have a poor prognosis and high mortality due to limited treatment options, recurrence and metastasis (5). Gastric cancer is a multi-factorial disease, and both environmental and genetic factors can influence its development (6). In recent years, although advances in surgery, radiation and chemotherapy regimens have helped to further reduce the incidence and mortality of gastric cancer, the overall 5-year survival rate is still only about 25% (7). Therefore, it is of great significance to further explore the molecular mechanism of the occurrence and development of gastric cancer and to find specific and sensitive biomarkers for early diagnosis, prognosis evaluation and effective treatment of gastric cancer.

In recent years, post-transcriptional modifications have been identified to play a key role in various physiological and pathological processes due to continuous advances of high-throughput sequencing technologies (8, 9). So far, more than 170 types of RNA modifications have been found in various RNA molecules (10). RNA modifications play an important role in the regulation of gene expression. Among them, RNA methylation has a variety of biological properties, including N6-methyladenosine (M6A) (11), 2-O-dimethyladenosine (M6Am) (12), N1-methyladenosine (M1A) (13), 5-methylcytosine (M5C) (14) and 7-methylguanosine (M7G) (15). N7-methylguanosine (M7G) is an important epigenetic modification and one of the most common RNA modifications. m7G modifications actively participate in physiological and pathological processes by affecting the metabolism of various RNA molecules such as messenger RNA (mRNA), ribosomal RNA (rRNA), microRNA (miRNA) and transfer RNA (tRNA) (10). There is increasing evidence that abnormal expression of m7G is closely associated with tumor development and is involved in a variety of tumor-related biological activities (16, 17).

Tumorigenesis involves a range of genetic variants, including single nucleotide mutations (SNV) and copy number variants (CNV). SNV, also known as SNP (single nucleotide polymorphism), as the most common type of genetic variation, refers to the variation of a single nucleotide that occurs at a specific location in the genome (18). Single nucleotides can be changed, deleted or inserted into a polynucleotide sequence (19). CNV is defined as the variation of DNA fragments in the human genome with copy numbers ranging from 1kb to several Mb, including DNA fragment deletions, insertions, duplications and compound multipoint variants (20, 21). CNV and SNV are not only important sources of human genetic diversity, but also play an important role in the occurrence and development of tumors, which are considered to be the key factors of tumor genetic variation (22–24).

In this study, we first analyzed the somatic mutations of m7G-related genes (m7GRGs) in TCGA- gastric cancer (STAD) patients. Next, we integrated TCGA and GSE15459 gastric cancer data to classify 567 patients into three different m7G subtypes based on prognostic m7GRG expression. At the same time, the signal pathway, immune cell infiltration and tumor microenvironment of different subtypes were analyzed. Then, GC patients were further classified into three genetic subtypes again according to the prognostic differentially expressed genes (DEGs) among the three subtypes. Finally, we predicted patients’ OS by constructing the m7GRG_score model and a nomogram. In addition, we analyzed the mutation, tumor microenvironment (TME), microsatellite instability (MSI) and drug sensitivity in high and low m7GRG_score groups. Also, microRNAs and lncRNAs related to model genes were explored.

## Material and methods

### Data sources and processing

**Figure 1:**
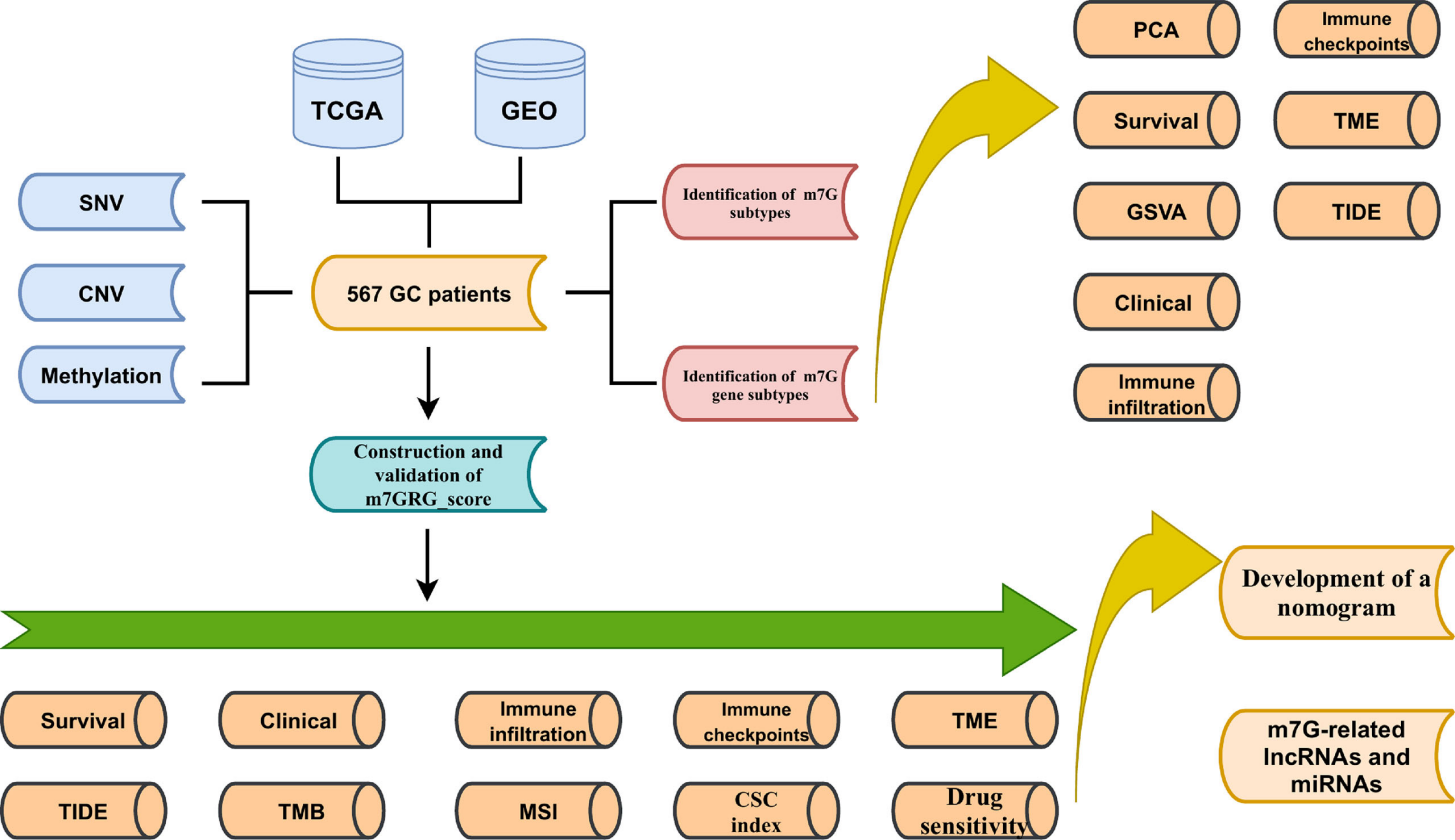
Schematic diagram of the flow of the study.

**Figure 1** shows the main flow of our work. Gene expression data (FPKM), relevant clinical information, CNV and SNV data for gastric cancer were downloaded from The Cancer Genome Atlas (TCGA). Meanwhile, the GSE15459 cohort data was extracted from Gene Expression Omnibus (GEO). Then the FPKM values of TCGA-STAD were converted to TPM values. Then, the two data sets were merged by normalizing the quartiles and removing the batch effect by the “Combat” algorithm. Thirty-eight m7G methylation-related genes were obtained from the known literature (25)and GO (http://www.gseamsigdb.org/gsea/login.jsp). Information on TCGA and GSE15459 GC patients is shown in **Table S1**. m7GRGs are presented in **Table S2**.

### Genetic characteristics

First of all, we analyzed the differential expression of m7GRGs between 375 GCs and 32 normal samples in TCGA-STAD using the limma package. The Maftools package was used to analyze mutations of m7GRGs and the top 3 genes with the highest percentage of mutations were selected to analyze whether gene mutations were associated with the expression of m7GRGs. Also, we analyzed the relationship between gene mutations and GC prognosis in the Gene Set Cancer Analysis (GSCA) database. In addition, the methylation levels and CNV changes of m7GRGs, and the correlation between these two and GC prognosis were also analyzed.

### Identification of m7G subtype

Thirteen prognostically relevant m7GRGs were identified by univariate COX and Kaplan-Meier survival analysis of m7GRGs. Subsequently, the 567 GC samples were typed based on the expression of these 13 genes using the ConsensusClusterPlus package. The effect of typing was assessed by principal component analysis (PCA). Survival package was used to analyze the survival of different subtypes. We also analyzed the correlation between the clinicopathological features of the three subtypes. To explore the role of different subtypes in GC, we used GSVA package to further compare the potential pathways between subtypes. CIBERSORT algorithm was used to analyze the difference of immune cell infiltration among different subtypes. The ESTIMATE algorithm was used to calculate the TME score. Additionally, we analyzed the expression of immune checkpoints between subtypes.

### Classification of genetic subtypes

To fully explore m7GRGs-related subtypes, we further analyzed differentially expressed genes (DEGs) among the three subtypes. Then, univariate cox analysis was performed on the DEGs, resulting in 79 prognostic differential genes. Based on the expression of these genes, the 567 GC samples were genotyped again. Finally, we analyzed the survival time, clinicopathological characteristics, and expression of m7GRGs in patients with different genetic subtypes.

### Construction of the prognostic model (m7GRG_score)

First, all GC samples were randomly divided into train (n=282) and test (n=281) groups in a ratio of 1:1. The Train group was used to construct the prognostic model, and the Test group was used to verify the accuracy of the model. Based on 79 subtype-associated prognostic DEGs, Lasso and multifactorial COX regression analysis was carried out. Five risk genes were ultimately identified and the m7GRG_score formula was obtained. According to the risk score formula, each GC patient in the Train group is granted a score. The patients were then sorted into high- and low- m7GRG_score groups based on the median scores. Similarly, the GC samples in the Test group were separated into high- and low- risk groups according to the m7GRG_score formula. Subsequently, Kaplan-Meier survival analysis was used to compare the overall survival (OS) of patients in the high and low m7GRG_score groups. To validate the accuracy of the model, the timeROC package was used to calculate the AUC of patients with 1-, 3-, 5-, and 7-year OS. higher AUC represent better predictive performance. The survival dynamic diagram visually shows the relationship between m7GRG_score and survival state. To further confirm the applicability of the m7GRG_score, we performed a stratified analysis of patients with different ages, genders, and stages. We also analyzed the relationship between different subtypes and genetic subtypes with m7GRG_score. To investigate whether m7GRG_score correlates with clinicopathological features, we analyzed the age, sex, and stage of patients.

### Immunocorrelation, tumor mutation burden, MSI, tumor stem cell index and GSEA analysis

The TME is the site of tumor cell growth, in which immune cells are an important factor influencing tumor progression. Therefore, we analyzed the immune cell infiltration and TME in the high- and low-m7GRG_score groups. At first, the infiltration differences of immune cell subpopulations between the two groups were analyzed by CIBERSORT algorithm. In parallel, the expression of immune checkpoint genes was also evaluated. Secondly, the ESTIMATE algorithm was taken to calculate immune scores, stromal scores, estimated scores and tumor purity for high - and low - risk groups. To further explore the differences in immune status between the different m7GRG_score groups, we analyzed the immune dysfunction score, immune exclusion score, and tumor immune dysfunction and exclusion (TIDE) score (http://tide.dfci.harvard.edu/).

The Maftools package was adopted to analyze gene mutations in different risk groups. The correlation between m7GRG_score and TMB was analyzed and presented by ggpubr and reshape2 packages. To investigate whether TMB affects the prognosis of patients, we divided patients into high and low TMB groups for survival analysis, based on median TMB. At the same time, combined with m7GRG_score, the patients were divided into four groups for survival difference analysis. The corrplot and circlize packages were used to detect whether immune cell changes affect TMB. Next, we compared the MSI status of different m7GRG_score groups. Tumor stem cells are essential for the development of tumors. Therefore, we conducted correlation analysis between CSC and m7GRG_score using two indicators. Meanwhile, to examine whether there is crosstalk between m6A and m7GRG_score, we performed correlation analysis between m7GRG_score and m6A-related genes. In addition, the relevant functional pathways of both groups were studied according to the c2.cp.kegg.v7.5.1symbols.gmt gene set using GSEA software.

### Construction of a nomogram

We Screened independent prognostic factors affecting OS in GC patients by univariate and multifactorial COX analysis of clinical characteristics and m7GRG_score. A nomogram was then constructed to improve the clinical applicability of the model based on independent prognostic factors. Nomogram was used to predict the 1-, 3-, 5-and 7-year survival rates of patients. At the same time, the calibration curve was used to test the prediction performance of nomogram.

### Drug efficacy assessment and CeRNA regulatory network

We evaluated the response of different m7GRG_score groups to diverse drugs. Firstly, pRRophetic package was used to predict the sensitivity of high and low risk groups to some common CG chemotherapeutic drugs (indicated by IC50 value). Secondly, we downloaded the IPS scores of CTLA4 and PD1 of GC patients from TCIA (https://tcia.at/home) to compare the immunotherapy effects of different m7GRG_score groups. Also, we compared the sensitivity of m7GRG to a variety of commonly used antitumor drugs. Finally, the sensitivity of m7GRG to non-immunotherapeutic agents was observed using the GSCA database (http://bioinfo.life.hust.edu.cn/GSCA/#/). To further delve into the possible mechanisms of m7G, we explored the upstream microRNAs and LncRNAs of five risk genes. First, the microRNAs related to each risk gene were queried in the starbase database (https://starbase.sysu.edu.cn). Then, correlation analysis was performed using the cor function to find the microRNAs with the highest negative correlation. Finally, the correlated lncRNAs of microRNAs were searched and the LncRNAs with negative correlation coefficients were screened. Visualization of the regulatory network is made in the Cytoscape software.

### Statistical analysis

All analyses were done using R software (version 4.1.1).

## Results

### Expression and mutation analysis of m7G-related genes in GC

**Figure 2:**
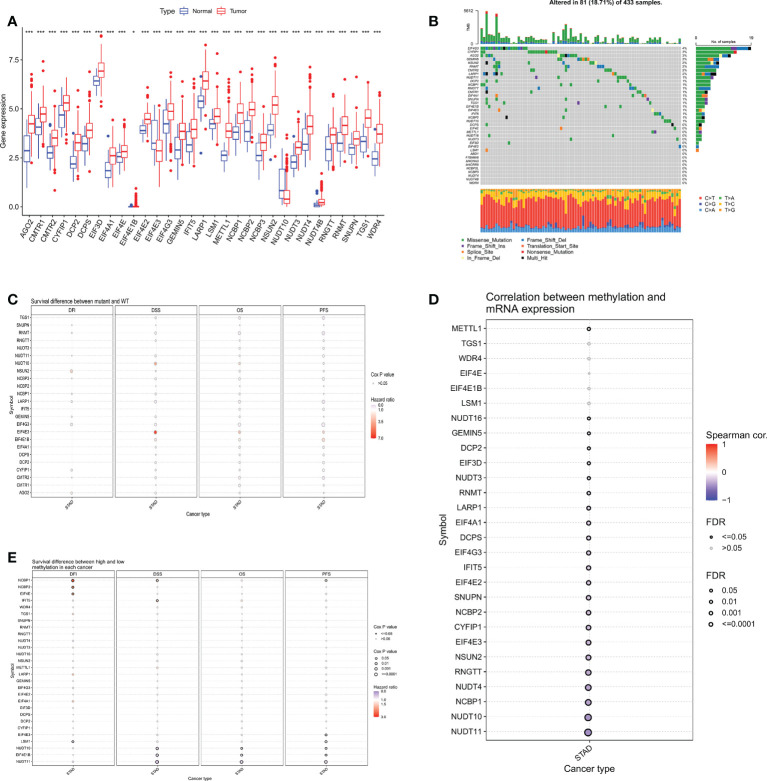
**(A)** Differential expression analysis of 31 m7GRGs in GC and normal tissues. **(B)** Mutation frequency of m7GRGs in GC patients in the TCGA-STAD cohort. **(C)** Relationship between mutations in m7GRGs and patient prognosis. Association of methylation levels of m7GRGs with gene expression **(D)** and patient prognosis **(E)**. m7GRGs, m7G-related genes; GC, Gastric cancer; STAD, Stomach adenocarcinoma; TCGA, The Cancer Genome Atlas. *P < 0.05 and ***P < 0.001.

The expression of 38 m7GRGs was analyzed in TCGA gastric cancer (n=375) and paraneoplastic tissues (n=32). The results as shown in the box plot, there were 31 m7GRGs with statistically significant differences in expression between the GC and normal groups (**Figure 2A**). In order to explore the genetic mutations of m7GRG in GC, we analyzed the somatic mutations of 38 m7GRGs. The results showed that the total mutation rate was 18.71%. Among them, the top three genes with the highest mutation frequency were EIF4G3, CYFIP1 and AGO2 (**Figure 2B**). To investigate whether the mutation rate was related to m7GRG expression, we selected the top 3 genes with the highest mutation rate and compared m7GRG expression between the mutant and wild type. The results showed that the expression of LSM1 in EIF4G3 mutant was higher than that in wild type (**Supplementary Figure S1A**). Mutant CYFIP1 had higher expression of GEMIN5 and EIF3D (**Supplementary Figure S1B**). SNUPN, LSM1, EIF4E, and DCPS expression were higher in the mutant AGO2 (**Supplementary Figure S1C**). This suggested that mutations were associated with gene expression, but only in a few genes. In addition, we explored the relationship between m7GRGs mutations and patient prognosis using cox analysis of the GSCA database. The results revealed that m7GRGs mutations did not affect the prognosis of patients, as the Cox P values were all >0.05 (**Figure 2C**). Meanwhile, we also analyzed the correlations of methylation levels of m7GRGs with gene expression and patient prognosis *via* the GSCA database. The expression of 23 m7GRGs was negatively correlated with the level of methylation (FDR < 0.05, **Figure 2D**). Hypermethylation of LSM1, NUDT10, EIF4E1B, NUDT11, NCBP1, NCBP2 and EIF4E was associated with poorer prognosis (**Figure 2E**). It is speculated that higher methylation levels of some m7GRGs may lead to lower gene expression and poorer prognosis.

**Figure 3:**
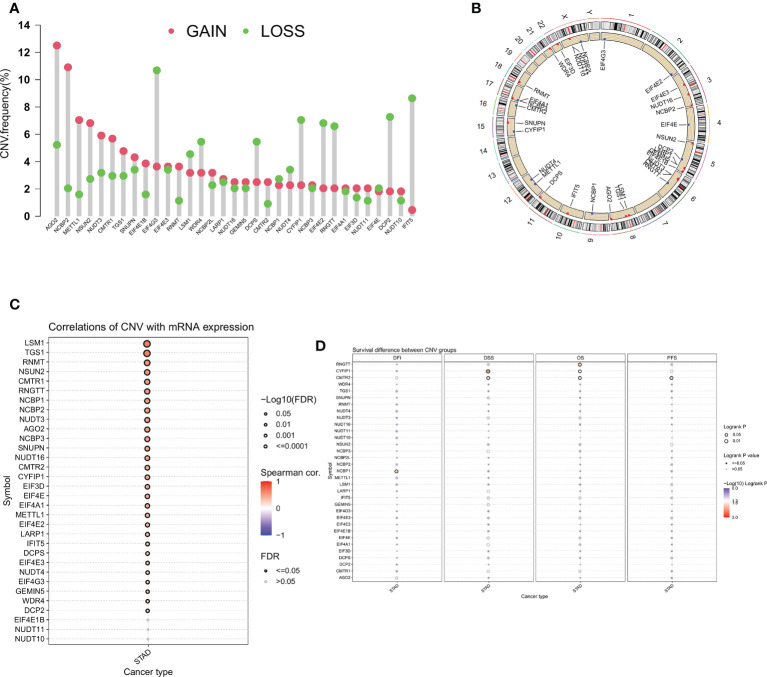
**(A)** Frequency of CNV change for m7GRGs. Red dots indicate CNV increase and green dots indicate CNV decrease. **(B)** The location of CNV alterations of m7GRGs on 23 chromosomes. Association of CNV alterations in m7GRGs with gene expression **(C)** and patient prognosis **(D)**. CNV, Copy number variation.

Next, we analyzed the CNV of m7GRGs and found that CNV alterations were prevalent in most m7GRGs. Among them, there were extensive CNV increases in AGO2, NCBP2, METTL1, and NSUN2, while CNV was reduced in EIF4E3, CYFIR1, EIF4E2, RNGTT, DCP2, and IFIT5 (**Figure 3A**). **Figure 3B** displays the location of CNV alterations in m7GRGs on their respective chromosomes. The same approach as above was used to explore the association of CNV changes in m7GRGs with gene expression and patient prognosis. The results showed that the expression of 29 m7GRGs was positively correlated with CNV levels (FDR < 0.05, **Figure 3C**). However, only 3 genes (RNGTT, CYFIP1, CMTR2) with increased CNV were associated with poor prognosis (**Figure 3D**). It is suggested that the CNV level of m7GRGs is positively correlated with gene expression, but it may not be related to the prognosis of patients.

### Identification of subtypes of GC patients based on m7GRGs

**Figure 4:**
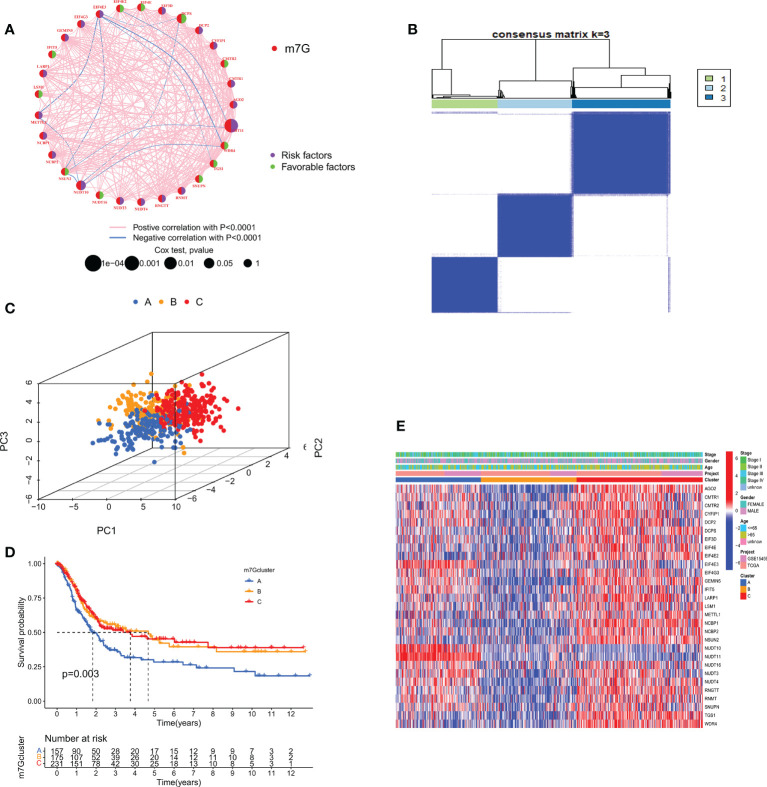
**(A)** Interaction between m7GRGs in GC. Connected lines indicate the existence of interactions between m7GRGs, and the thickness of the line indicates the strength of the association between m7GRGs. Pink and blue represent negative positive and negative correlations, respectively. **(B)** Consensus clustering analysis of three clusters (k = 3). **(C)** PCA showed significant differences among the three subtypes. **(D)** Kaplan-Meier curves for survival differences among the three subtypes. **(E)** Heat map of the analysis of differences among the three subtypes and correlation analysis between subtypes and clinicopathological features. PCA, Principal component analysis.

We combined TCGA-STAD with GSE15459 data and obtained a total of 567 GC patient data for further analysis. Through univariate Cox regression **Table S3** and Kaplan-Meier survival analysis (**Supplementary Figure S2**) of 31 m7GRGs, 13 m7GRGs related to prognosis were obtained. A comprehensive analysis of the interactions between m7GRGs and their prognostic value in gastric cancer is shown in the m7G methylation network diagram (**Figure 4A**). Meanwhile, we searched and downloaded the immunohistochemical results of some m7G regulatory genes expressed in adjacent normal and gastric cancer tissues from The Human Protein Atlas database (http://www.proteinatlas.org/), as shown in **Supplementary Figure S3**. To fully understand the mechanism of m7GRG in gastric carcinogenesis, we performed consensus cluster analysis on 567 gastric cancer samples based on the expression profiles of 13 prognostic m7GRGs and identified three subtypes, namely subtypes A, B and C (**Figure 4B**). To validate the clustering results, we further examined the three clusters with PCA. PCA plot demonstrated that there were significant differences among the three subtypes and could well distinguish GC patients (**Figure 4C**). Kaplan-Meier curves showed that patients with subtype A had a lower OS than patients with subtype B and C, suggesting that subtype A had a worse prognosis (log-rank test, p = 0.003; **Figure 4D**). Then, we analyzed the expression differences of m7GRGs between different subtypes and their correlation with some clinicopathological features (**Figure 4E**). The results showed that there was a significant difference between the expression of m7GRGs and age. Unfortunately, it has no correlation with other clinical features.

### Analysis of GSVA and TME for distinct subtypes

**Figure 5:**
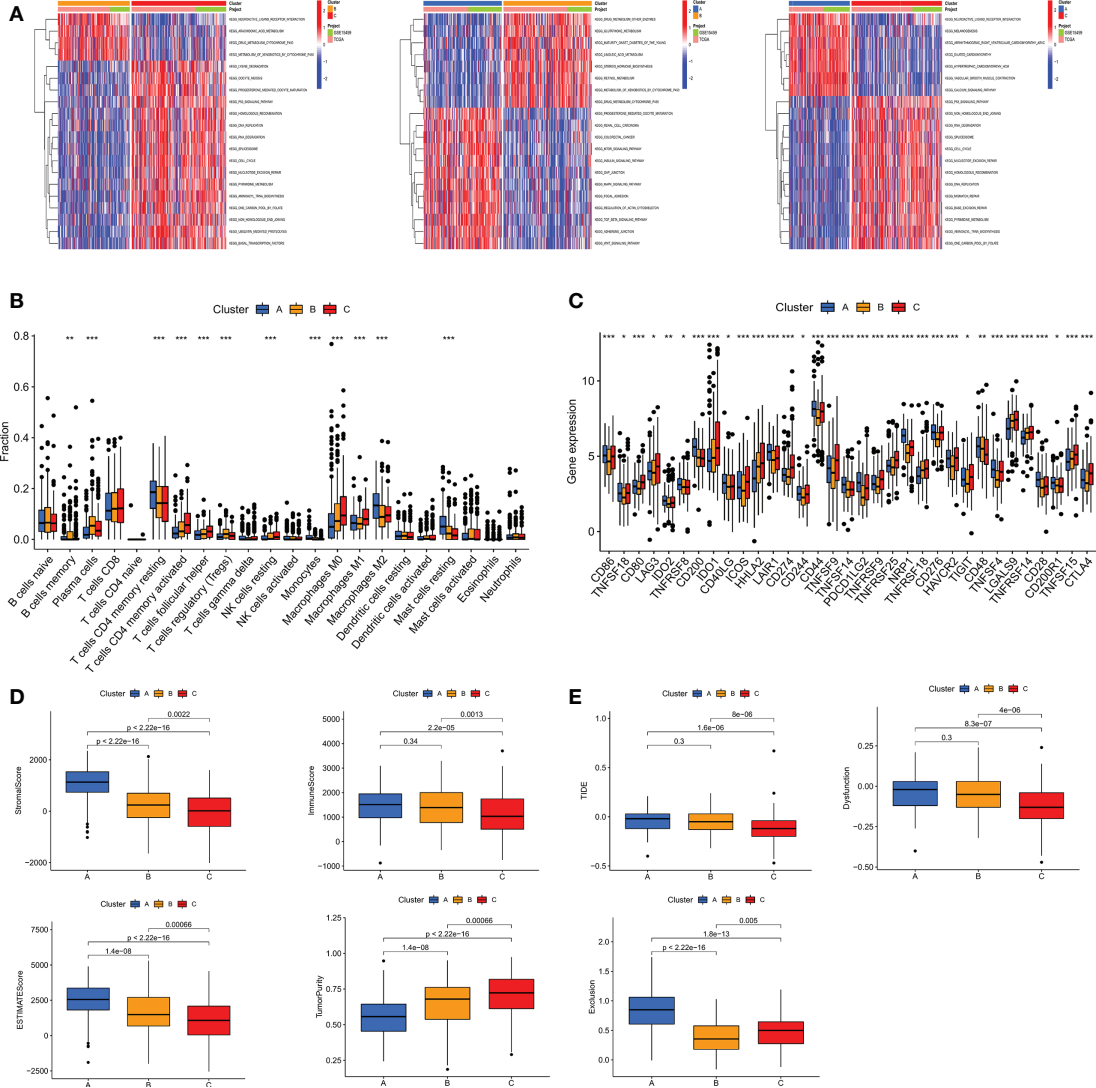
**(A)** GSVA pathway enrichment analysis between subtypes An and B, subtypes An and C, and subtypes B and C. **(B)** Differences in immune cell infiltration among different subtypes of GC were analyzed using the CIBERSORT algorithm. **(C)** Analysis of immune checkpoint expression among different subtypes. **(D)** The immune score, stromal score, ESITIMATE score and tumor purity among the three subtypes were calculated by ESITIMATE algorithm. **(E)** Immune dysfunction score, exclusion score and TIDE score among the three subtypes. TIDE, Tumor immune dysfunction and exclusion. *P < 0.05, **P < 0.01 and ***P < 0.001.

TME, as the “soil” for the growth of tumor cells, contains a variety of immune cells and stromal cells, such as endothelial cells and fibroblasts (26).TME creates a favorable environment for the inhabitation and interaction of tumor cells and other cells in their vicinity. At the same time, a growing body of data suggests the importance of TME in guiding patients in their choice of treatment (27). To further discover the potential mechanisms between the different subtypes, we performed GSVA pathway enrichment analysis for each two of the three subtypes in turn. The results revealed that some classical tumor-related signal pathways, such as WNT, TGF-β, mTOR and MAPK, were significantly enriched in cluster A. Cluster B is more enriched in metabolism-related signaling pathways like retinol metabolism, linoleic acid metabolism, glutathione metabolism and arachidonic acid metabolism. The cell cycle-related pathways are more tightly associated with cluster C (**Figure 5A**). To investigate the role of m7GRGs in the tumor immune microenvironment of GC, we analyzed the infiltration of 22 human immune cell subsets in different subtypes using the CIBERSORT algorithm (**Figure 5B**). The results confirmed that there were significant differences in immune cell infiltration among the three subtypes. Compared with the other two subtypes, subtype A had more infiltration of resting CD4+ memory T cells, M2 macrophages and resting mast cells, while less infiltration of memory B cells, plasma cells, CD8+T cells, activated CD4+ memory T cells, follicular helper T cells, regulatory T cells (Treg), resting NK cells, M0 macrophages and M1 macrophages. Meanwhile, we evaluated the expression of immune checkpoints. The results showed that there were obvious differences in the expression of several immune checkpoints among the three subtypes (**Figure 5C**). Since there were significant differences in immune cell infiltration, we further evaluated the TME scores of the three subtypes with ESTIMATE algorithm. The results showed that subtype A had higher immune score, stromal score, ESITIMATE score and lower tumor purity (**Figure 5D**), which indicated that the content of immune cells and stromal cells in subtype A was relatively high. It is well known that higher TIDE scores are associated with poorer immunotherapy and shorter survival. Therefore, the TIDE score will facilitate the screening of patients who are more suitable for immunotherapy. Subtype A had a higher TIDE score than subtype B and C (**Figure 5E**). This corresponds to the previous conclusion that subtype A has poorer OS.

### Identification of gene subtypes based on prognostic DEGs

**Figure 6:**
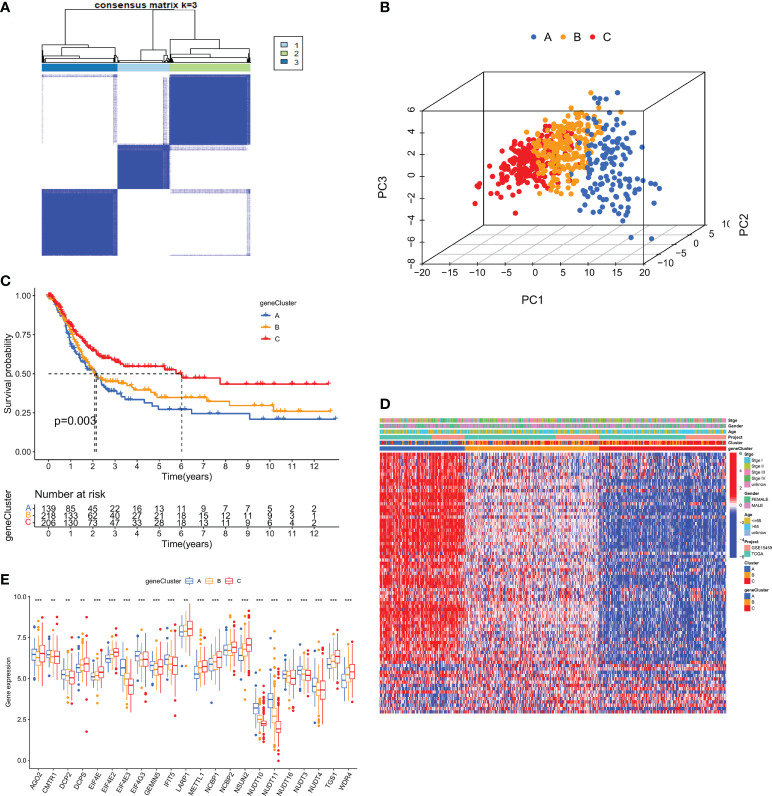
**(A)** Consensus clustering matrix for genotyping of GC patients based on 79 prognostic DEGs. **(B)** PCA Analysis for testing clustering effect. **(C)** Kaplan-Meier curves for OS of the three gene subtypes. **(D)** The correlation heat map between clinicopathological features and three gene subtypes. **(E)** Differential expression analysis of m7GRGs in three genetic subtypes. DEGs, Differentially expressed genes; OS, Overall survival. **P < 0.01 and ***P < 0.001.

To investigate the typing of gastric cancer in depth, we further analyzed the DEGs among the three m7G subtypes and obtained 194 subtype-related DEGs. The DEGs were then subjected to univariate cox analysis, and 79 DEGs associated with prognosis were acquired. 567 gastric cancer samples were genotyped again by consensus clustering algorithm based on the expression of prognostic DEGs. Likewise, the results showed that k=3 was the more desirable genotyping result, that is, the samples were divided into geneCluster A, geneCluster B and geneCluster C (**Figure 6A**). The PCA plot also further proved that the genotyping had a better clustering effect (**Figure 6B**). Importantly, we found that the majority of samples in genotype A were classified as subtype A. Similarly, samples in genotypes B and C were strongly related to subtypes B and C, respectively (**Figure 6D**). Kaplan-Meier curves suggested that patients with genotype A had the worst OS, while those with genotype C had the greatest OS (log-rank test, p = 0.003; **Figure 6C**). This finding was consistent with the result of survival analysis of the m7G subtype. In addition, the expression of m7GRGs in the three gene subtypes showed that there were 22 m7GRGs expression differences were statistically significant (**Figure 6E**).

### Construction and validation of the prognostic risk model (m7GRG_score)

Based on the 79 subtype-related prognostic DEGs obtained above, we first performed lasso regression analysis and selected the 9 genes corresponding to the lambda value with the lowest cross-validation error (**Supplementary Figure S4A**). Then a multivariate cox analysis was performed on 9 genes, and finally 5 risk genes (APOD, COL10A1, GPX3, KIAA1324, CTSV) were obtained. The 5 risk genes were used to construct the prognostic m7GRG_score. The coefficients for each risk gene in the model are shown in **Table 1**. The m7GRG_score formula is as follows:

**Table 1:** The five risk genes and their coefficients involved in the construction of the model.

symbol	coef
APOD	0.0796304485112953
COL10A1	0.119584867343104
GPX3	0.207322154707039
KIAA1324	-0.0895063827152558
CTSV	0.185159064583688

Risk score = (0.0796304485112953* expression of APOD) + (0.119584867343104* expression of COL10A1) + (0.207322154707039* expression of GPX3) + (0.185159064583688* expression of CTSV) + (-0.0895063827152558* expression of KIAA1324)

**Figure 7:**
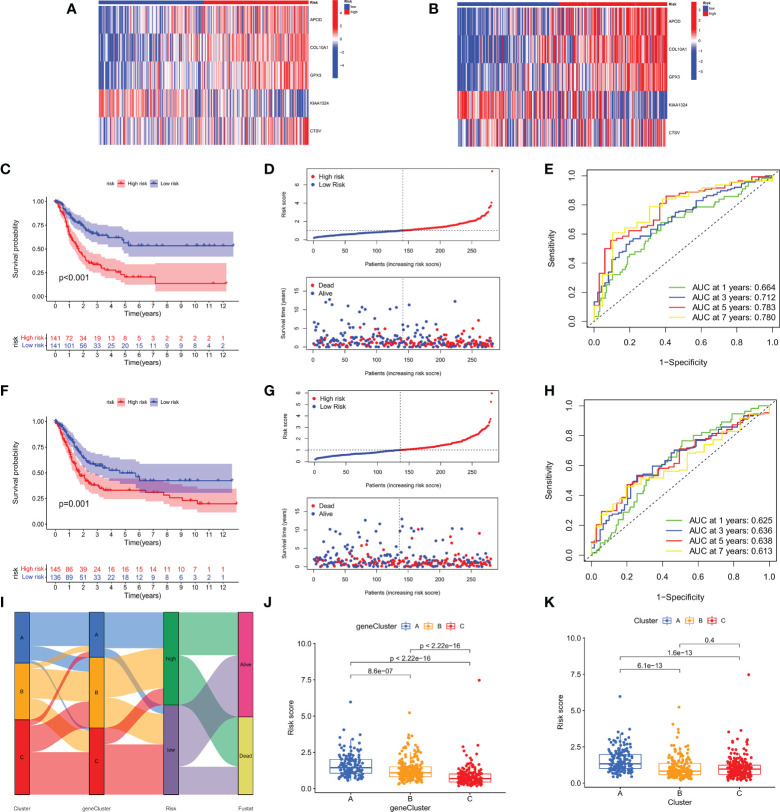
Heat map of the expression of the five risk genes in the high- and low- risk groups in Train **(A)** and Test groups **(B)**. OS analysis **(C)**, Risk score distribution and patient survival status **(D)**, and ROC analysis of predicted 1-, 3-, 5-, and 7-year survival according to m7GRG_score **(E)** in the Train group. Survival analysis **(F)**, risk curve **(G)** and ROC analysis **(H)** in the Test group. **(I)** Alluvial diagram of the distribution of patients with different m7G subtypes, genetic subtypes, m7GRG_score and survival outcomes. **(J)** Differences in m7GRG_score among the three genetic subtypes. **(K)** Differences in m7GRG_score of the three m7G subtypes.

All GC patients were divided into train group (n = 282) and test group (n = 281) in a 1:1 ratio. To assess the prognostic value of the risk model, we first calculated the m7GRG_score for each patient according to the risk formula. Patients were then divided into high- and low- m7GRG_score groups based on the median risk score of the Train group. In the same way, patients in the Test group can be divided into high- and low-risk groups. **Figures 7A, B** present heat maps of the expression of the five risk genes in the Train and Test groups, respectively. Survival analysis confirmed that OS was significantly worse in patients with high m7GRG_score than in patients with low m7GRG_score (p<0.01, **Figure 7C**). As shown in **Figure 7D**, as the risk of GC patients increases, the number of deaths increases gradually. The ROC curve shows that the area under the curve (AUC) of 1-, 3-, 5-and 7-year is 0.664, 0.712, 0.783 and 0.780, respectively (**Figure 7E**). The above results revealed that the risk model had a favorable discriminatory ability for the prognosis of gastric cancer patients. To further test the accuracy and reliability of the model, we used the Test group to validate the model. The results showed a good validation efficiency (**Figures 7F–H**). **Figure 7I** displays the distribution of patients in three m7G subtypes, three genetic subtypes, and two risk groups. In addition, we grouped patients by age (≤60 and >60 years), sex (female and male) and stage (stages I-II and III-IV) according to a risk score formula, followed by survival analysis. The results showed that patients with high m7GRG_score had a worse prognosis, which further demonstrated the applicability of the model (**Supplementary Figure S4B-D**). Through the analysis of the risk scores of three m7G subtypes, it is found that the risk score of subtype A was significantly higher than that of subtypes B and C, which corresponded to the worse OS of subtype A (**Figure 7J**). Similarly, the risk score of gene subtype A was significantly higher than that of the other two gene subtypes, which was consistent with the worse survival of gene subtype A (**Figure 7K**). Finally, to reveal whether m7GRG_score is related to clinicopathological factors, we compared the risk scores of patients with different ages, genders and stages, and found that there was a significant correlation between m7GRG_score and stages (**Supplementary Figure S4E**). Similarly, according to the risk model, we also scored patients in the same stage, and divided patients in the same stage into high and low m7GRG_score groups based on the median score. We then performed survival analysis, tumor mutational burden analysis (TMB) and microsatellite instability (MSI) analysis for both groups of patients. The results showed that in the same stage of patients, compared with the high-risk group, the low-risk group had longer survival, higher TMB and higher MSI-H ratio (**Supplementary Figure S5**). These results suggest that for patients in the same stage, patients with low scores have better survival, are more sensitive to immunotherapy and are more likely to benefit from immunotherapy.

### Analysis of immune cell infiltration, TME, TMB and MSI between high- and low- m7GRG_score groups

First, we utilized CIBERSORT algorithm to analyze the infiltration of 22 kinds of immune cell subsets between high- and low- m7GRG_score groups. The results showed that the main infiltrating immune cells in the high m7GRG_score group were M2 macrophages, monocytes and resting mast cells (**Supplementary Figure S6A**). Additionally, we also analyzed the link between immune checkpoints and risk models. The results suggested that 30 immune checkpoints were differentially expressed in the two groups, including CD80, CD86 and CTLA4 (**Supplementary Figure S6B**). Finally, we analyzed the TME scores of both groups using the ESTIMATE algorithm and found that the immune score, stromal score, and ESTIMATE score were higher, and the tumor purity was lower in the high-risk group (**Supplementary Figure S6C**). Also, the high-risk group was associated with higher TIDE scores (**Supplementary Figure S6D**).

**Figure 8:**
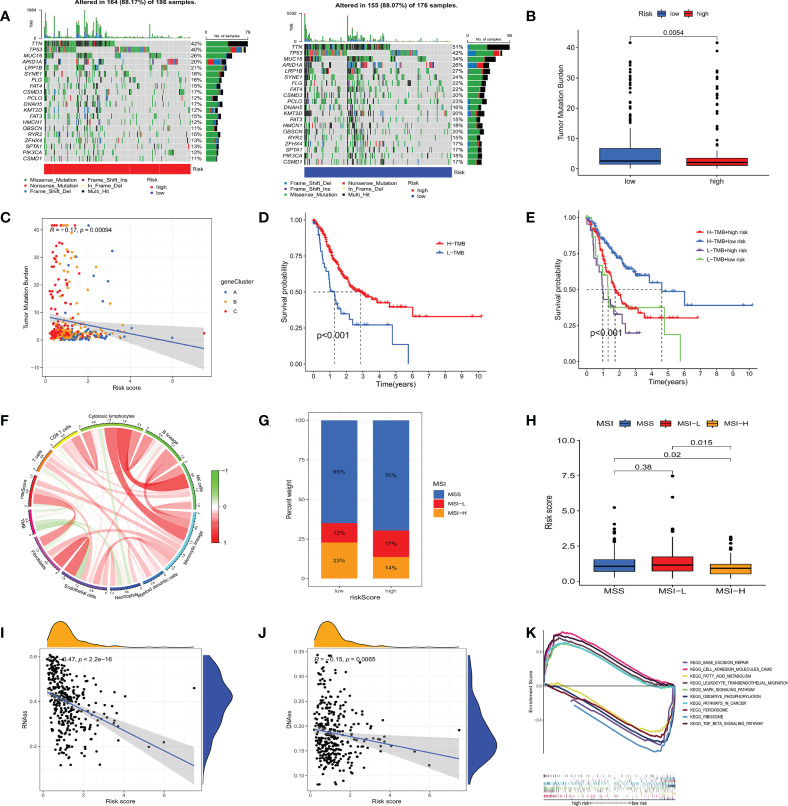
**(A)** Waterfall plots of somatic mutation characteristics in high- and low- risk groups. **(B)** The difference of TMB in different m7GRG _ Score groups. **(C)** Spearman correlation analysis between m7GRG _Score and TMB. **(D)** Survival analysis of high- and low- TMB groups. **(E)** Survival analysis of four groups of patients stratified by m7GRG _Score and TMB. **(F)** The relationship between TMB and immune cells. Red represents positive correlation and green represents negative correlation. The shade of color represents the degree of correlation. **(G, H)** Relationship between m7GRG _Score and MSI. **(I, J)** Relationship between m7GRG _Score and CSC index. **(K)** GSEA signaling pathway enrichment analysis of high- and low- m7GRG_score groups. TMB, Tumor mutation burden; MSI, Microsatellite instability; CSC, tumor stem cell; GSEA, Gene Set Enrichment Analysis.

To investigate the genomic mutations in the high and low m7GRG_score groups, we analyzed the somatic mutations in the two groups in the TCGA-STAD cohort separately. The results showed that the top three genes with the highest mutation frequencies in both groups were TTN, TP53 and MUC16 (**Figure 8A**). Growing evidence suggests that TMB serves as a valid predictive biomarker for immune checkpoint blockade therapy in some types of cancer (28, 29). This implies that patients with elevated TMB may be more sensitive to immunotherapy, which is helpful for clinical screening of immunotherapy population. We noted a higher TMB in the low m7GRG_score group, suggesting that patients in the low-risk group are more likely to be sensitive to immunotherapy (**Figure 8B**). Spearman correlation analysis showed that m7GRG_score was negatively correlated with TMB (**Figure 8C**). In addition, survival analysis of patients in the high and low TMB groups revealed that overall survival was worse in the low TMB group (**Figure 8D**), which may be related to the insensitivity of patients to immunotherapy. By combining TMB with the risk model, we found that patients in the low-risk+high-TMB group had the best prognosis and those in the high-risk+low-TMB group had the worst prognosis, which was consistent with our previous analysis (**Figure 8E**). We further explored the relationship between TMB and immune cells and found that TMB showed a significant negative correlation with Endothelial cells and neutrophils (**Figure 8F**). Previous studies have demonstrated that high microsatellite instability (MSI-H) can lead to accumulation of somatic mutations in tumor cells, increased neoantigen expression and enrichment of tumor infiltrating lymphocytes so that patients can benefit from immunotherapeutic agents (30). Correlation analysis showed that low m7GRG_score was significantly associated with MSI-H status, while high m7GRG_score was related to microsatellite stability (MSS) (**Figure 8G**). The identical result can be obtained in **Figure 8H**. These findings are consistent with the previous conclusion that the low-risk group is more sensitive to immunotherapy. By analyzing m7GRG_score with CSC index, we found that CSC index was negatively correlated with risk score (RNAs, R=-0.47, P<0.01; DNAs, R=-0.15, P<0.01) (**Figures 8I, J**). Subsequently, we performed GSEA analysis to identify signaling pathways in the high and low m7GRG_score groups that may be involved in regulating tumorigenesis. The results showed that some classical tumor-related signaling pathways and pathway in cancer were remarkably enriched in the high-risk group, while the low-risk group was more closely associated with some metabolism-related pathways (**Figure 8K**).

### Construction of nomogram

**Figure 9:**
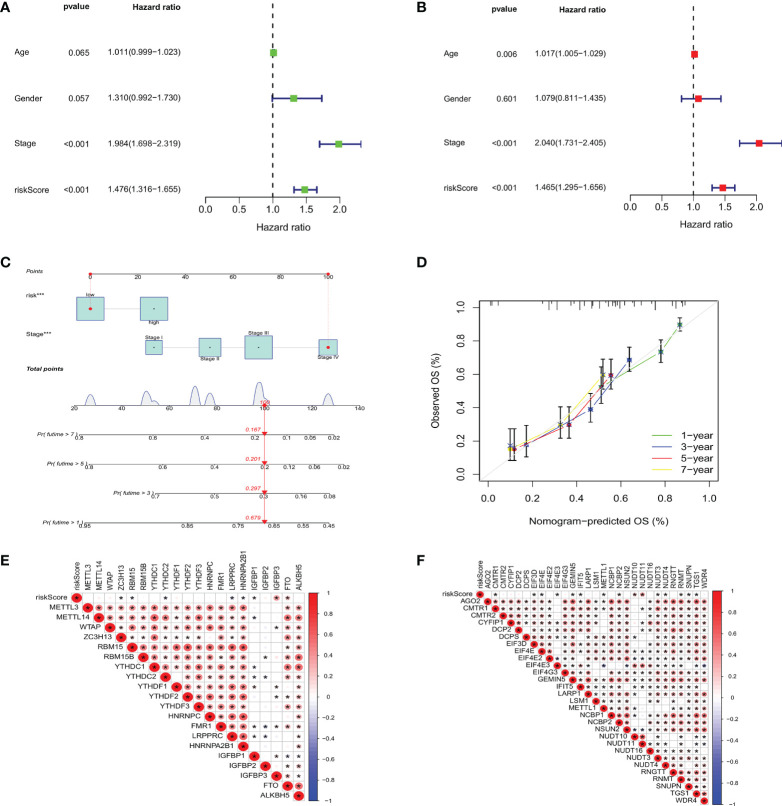
Based on univariate **(A)** and multivariate Cox analysis **(B)**, m7GRG_Score was determined to be an independent prognostic factor for GC patients. **(C)** nomogram for predicting 1 -, 3 -, 5-and 7-year OS in GC patients. **(D)** Calibration curves for nomogram with predicted 1-, 3-, 5-, and 7-year OS. Correlation analysis of m6A **(E)** and m7G **(F)** related genes with m7GRG _Score. *P < 0.05.

To investigate whether m7GRG_score is an independent prognostic factor for GC patients, we performed univariate and multifactorial Cox regression analyses on risk models and some clinical features, such as age, gender and stage. The results revealed that both risk model and stage were independent prognostic factors for GC patients (**Figures 9A, B**). To further strengthen the clinical application of the model, based on the above results, we constructed a nomogram containing the model and stage to predict 1-, 3-, 5-, and 7-year OS rates (**Figure 9C**). Moreover, the calibration plots showed that the survival time predicted by the model was highly agreement with the actual survival time (**Figure 9D**). It has been reported that METTL3 levels were elevated in gastric cancer tissues and enhanced the stability of heparin binding growth factor (HDGF) mRNA expression through m6A modification, which further induced gastric cancer cell glycolysis and promoted gastric cancer cell proliferation and liver metastasis (15). Therefore, we conducted correlation analysis of m6A and m7G-related genes with risk models, respectively. For m6A-associated genes, we found that risk scores were positively correlated with IGFBP3 and FTO (P<0.05) and negatively correlated with ZC3H13, RBM15B, YTHDC2, LRPPRC, HNRNPA2B1 and IGFBP1 (**Figure 9E**). Similarly, risk scores were also relevant to m7G-related genes (**Figure 9F**).

### Drug sensitivity analysis

**Figure 10:**
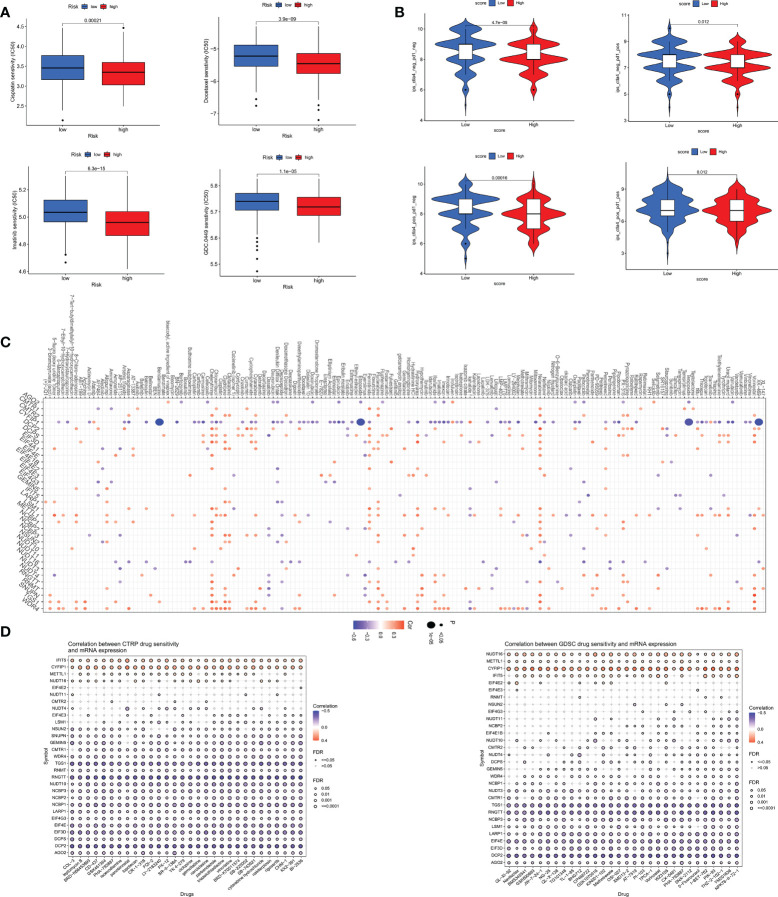
**(A)** Sensitivity analysis of high- and low- risk groups to four common GC therapeutic agents. **(B)** sensitivity analysis of high- and low- risk groups to immune checkpoint blocking therapy, such as anti-PD-1 and anti-CTLA4. Sensitivity analysis of m7GRGs to some antitumor drugs **(C)** and non-immunotherapeutic drugs **(D)**. PD-1, programmed death-1; CTLA4, Cytotoxic T lymphocyte-associated antigen-4.

We analyzed some GC chemotherapeutic agents commonly used in clinical to compare the sensitivity of high and low m7GRG_score groups to these drugs. The results showed that patients in the high m7GRG_score group had lower IC50s for Docetaxel, Cisplatin, Imatinib and GDC.0449, suggesting that high-risk patients are more sensitive to these four drugs (**Figure 10A**). We then compared the effect of high and low m7GRG_score groups on immune checkpoint PD-1 and CTLA4 blockade treatment. The results revealed that the low-risk group was more effective for anti-PD-1 or anti-CTLA4 or a combination of both (**Figure 10B**). **Figure 10C** demonstrated the sensitivity of m7GRGs to some antineoplastic drugs. At the same time, we also analyzed the sensitivity of m7GRGs to non-immunotherapeutic drugs (**Figure 10D**). In conclusion, these results suggest that m7GRGs are associated with drug sensitivity.

### Analysis of CeRNA regulatory network

**Figure 11:**
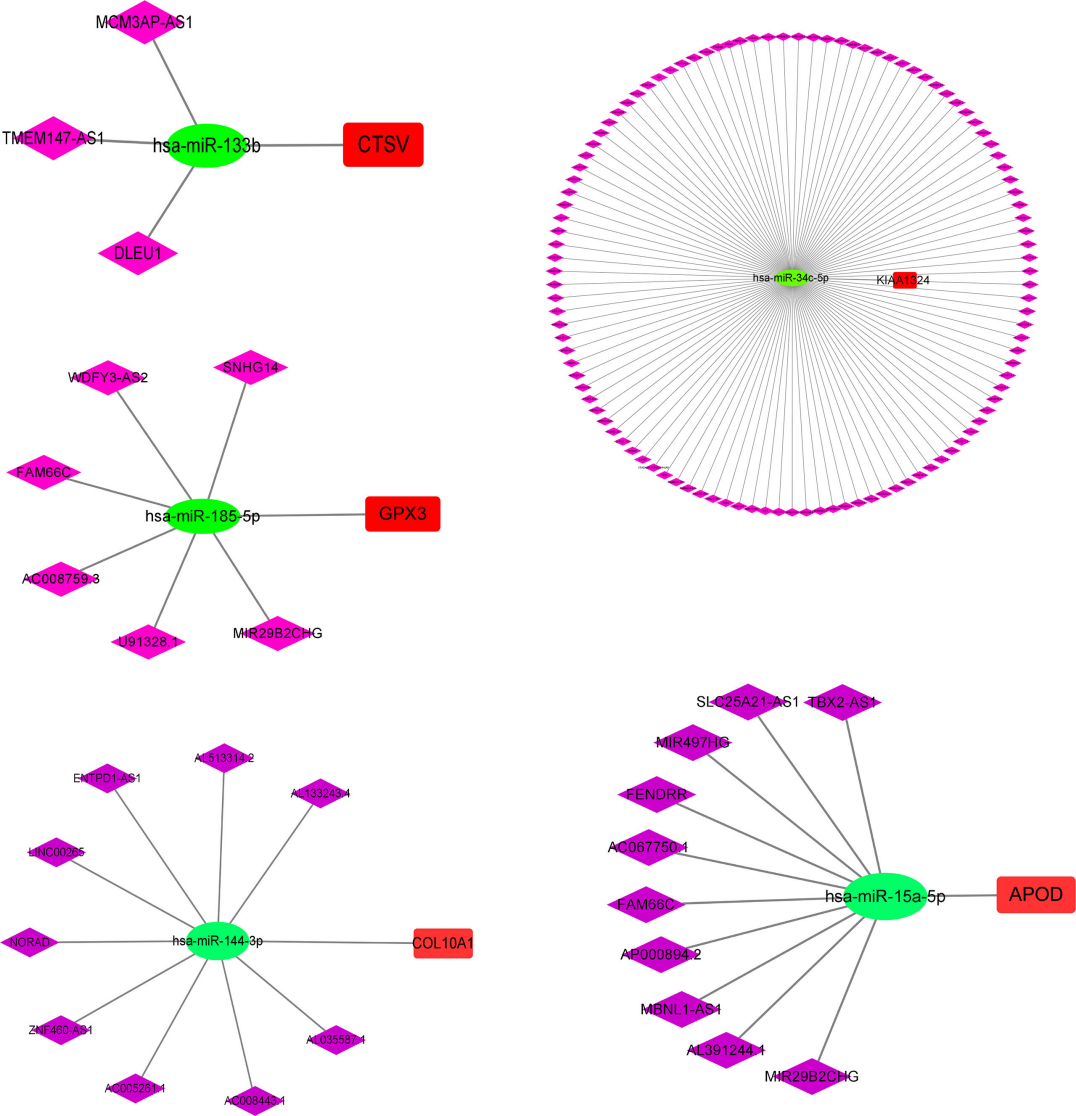
CeRNA Regulatory Network Analysis of five risk genes. CeRNA, competing endogenous RNAs.

To explore the mechanism of m7G in GC in depth, we further analyzed the upstream regulatory molecules of five risk genes. First, we looked for upstream miRNAs that might act on risk genes (mRNAs) in the starbase database. Then some miRNAs that were significantly negatively regulated with risk genes were selected by correlation analysis. Next, we searched for lncRNAs that were negatively correlated with microRNAs. The results showed that MCM3AP-AS1, TMEM147-AS1 and DLEU1 may competitively bind has-miR-133b, thereby attenuating the inhibitory effect of has-miR-133b on CTSV. Likewise, SNHG14, WDFY3-AS2, FAM66C, AC008759.3, U91328.1 and MIR29B2CHC might competitively bind has-miR-185-5p, thus weakening the inhibitory effect of has-miR-185-5p on GPX3. The regulatory relationships of CTSV, GPX3, KIAA1324, APOD and COL10A1 are presented in **Figure 11**, individually.

## Discussion

In recent years, as an important form of post-transcriptional modification, m7G has attracted the attention of more and more researchers. Many studies have proved that the abnormal regulation of m7G methylation at various RNA levels plays an important role in the occurrence and development of tumors. Peng et al. (31) found that m7G methyltransferase WD repeat domain 4 (WDR4) inhibits apoptosis by increasing the level of m7G methylation in hepatocellular carcinoma (HCC), thereby promoting HCC cell proliferation and metastasis. Chen et al. (32) demonstrated that the m7G methyltransferase METTL1 promotes the progression of head and neck squamous cell carcinoma by mediating aberrant translation regulated by tRNA N7-methylguanosine (m7G) modification. However, studies on the role of m7G modification in gastric cancer are relatively scarce. Therefore, in this paper, the role of m7G-related genes in gastric cancer was analyzed by bioinformatics approach. Li et al. (33) have also done related work, but our analysis is more in-depth and comprehensive than their study. First of all, compared with that literature, this study analyzed the somatic mutations of m7GRGs in GC patients, including SNV and CNV. At the same time, we further analyzed whether gene mutations were somehow associated with m7GRGs expression and patient prognosis. More importantly, based on 38 m7GRGs, we classified GC patients twice, which made the possible mechanism of m7GRGs in the occurrence and development of gastric cancer further explored. These are not analyzed by Li et al. In addition, we performed a more comprehensive comparison of patients in the high- and low-risk groups, including immune cell infiltration, TME scores, TMB, MSI, and drug sensitivity analysis. Finally, we also performed CeRNA regulatory network analysis for five risk genes. In conclusion, our study is a useful reference for exploring in depth the potential mechanisms of m7G in gastric cancer, searching for new molecular markers of GC prognosis and screening out the population most likely to benefit from immunotherapy.

This study mainly focused on typing GC patients and constructing a prognostic risk model from the perspective of m7G modification, and clarified the important role of m7GRG_score in survival status, immune cell infiltration, TME analysis, tumor mutation load and drug sensitivity, which allowed for a more accurate prognostic analysis of GC patients. First, we analyzed the somatic mutation and CNV data of GC in TCGA and compared m7GRG methylation and CNV changes with gene expression and patient prognosis. Then, we integrated the gene expression data and clinical data of GC patients from TCGA and GSE15459, and divided the patients into three subtypes according to the expression of 13 prognosis-related m7GRGs. Patients with subtype A had lower OS and higher TIDE scores than the other two subtypes. Meanwhile, several classic tumor-associated signaling pathways were significantly enriched in subtype A, such as WNT, TGF-β, mTOR and MAPK signaling pathways. There were also significant differences in immune cell infiltration, immune checkpoint gene expression and TME characteristics among the three subtypes. Based on the DEGs among the three subtypes, we identified three genetic subtypes. Samples in genetic subtypes A, B, and C were closely related to those in subtypes A, B, and C, respectively. As with subtype A, patients with genotype A had the worst OS.

In this study, we constructed a prognostic m7GRG_score consisting of five risk genes and validated its accuracy. The model was used to predict clinical outcomes and immunotherapy response in GC patients. Also, the model has good predictive power for the prognostic survival time of patients. The model demonstrated significant differences in prognosis, immune cell infiltration, TME, TIDE, mutations, TMB, MSI, drug sensitivity, and signaling pathways between patients in the high-risk and low-risk groups. Finally, we established a nomogram based on m7GRG_score and tumor stage to further improve the clinical application of the model.

Many studies have confirmed that macrophages are divided into two main subtypes based on their function, namely classically activated M1-type macrophages and alternatively activated M2-type macrophages (34). M1 macrophages mainly secrete pro-inflammatory cytokines, which promote inflammatory response, pathogen clearance and anti-tumor immunity, while M2 macrophages secrete anti-inflammatory cytokines and exert immunosuppressive phenotype, which is beneficial to tissue repair and tumor progression (35). As the major infiltrating immune cells in the tumor microenvironment, M2 macrophages have vital roles in regulating tumor cell proliferation, migration and invasion through the production of various factors, protein hydrolases and inhibitory immune checkpoint proteins (36, 37). Thus, in most tumor types, the number and density of M2 macrophages have a significant negative correlation with patient prognosis (38, 39). Consistent with the findings of previous studies, in the present study, M2 macrophage infiltration was significantly increased in the subtype A and high m7GRG_score groups, along with a poorer prognosis in both groups.

Because early symptoms of GC are insidious and not easily detected, and the prognosis of patients with advanced disease is extremely poor, GC remains a major problem that endangers human health. In recent years, the rapid development of immunotherapy has brought hope. Immunotherapy based on immune checkpoint inhibitors (ICI), such as monoclonal antibody therapy against PD-1, PD-L1 and CTLA4, is being widely studied and applied to clinical treatment. Currently, nivolumab and Pembrolizumab, monoclonal antibodies against PD-1, are approved for third-line treatment of PD-L1-positive advanced gastric cancer (40–42). On April 16, 2021, FDA approved nivolumab combined with chemotherapy for first-line treatment of advanced or metastatic gastric cancer (43). In addition, several biomarkers, notably MSI, PD-L1, human epidermal growth factor receptor 2 (HER2), vascular endothelial growth factor (VEGF) and TMB, have significant predictive value in GC immunotherapy and are increasingly being used to identify populations most likely to benefit from immunotherapy and targeted therapy (44, 45). In the present study, we observed that the low m7GRG_score group was remarkably associated with MSI-H and higher TMB and was more sensitive to anti-PD-1 and anti-CTLA4 treatments. It is suggested that patients in the low m7GRG_score group may respond to immune checkpoint blockade therapy.

Unfortunately, however, our study also has some shortcomings. Firstly, the data we analyzed were all sourced from public databases. Secondly, the sample size we collected was not large enough. Therefore, the conclusions drawn in this paper require additional samples to verify their accuracy, as well as *in vivo* and *in vitro* experiments to further explore the role and mechanisms of m7G-related genes in GC.

## Data availability statement

The original contributions presented in the study are included in the article/**Supplementary Material**. Further inquiries can be directed to the corresponding author.

## Author contributions

XL, HD, XP, XZ, LC, YW, ZH, YJ, ZZ, and YZ conceived the present study. XL, HD, LC, YW, ZH, YJ, ZZ, and YZ performed the statistical analysis and wrote the draft. XP and XZ critically revised the work. All authors contributed to the article and approved the submitted version.

## Funding

This study was supported by grants from the National Natural Science Foundation of China (82072707), Scientific research program of Shanghai Municipal Commission of science and technology (19411970700 and 20Y11909400) and the Changhai Hospital 234 Project (2019YXK019 and 2020YXK029).

## Conflict of interest

The authors declare that the research was conducted in the absence of any commercial or financial relationships that could be construed as a potential conflict of interest.

## Publisher’s note

All claims expressed in this article are solely those of the authors and do not necessarily represent those of their affiliated organizations, or those of the publisher, the editors and the reviewers. Any product that may be evaluated in this article, or claim that may be made by its manufacturer, is not guaranteed or endorsed by the publisher.

## Supplementary material

Supplementary Figure 1 The relationship between the mutation status of EIF4G3 (**A**), CYFIP1 (**B**), AGO2 (**C**) and the expression of M7G related genes.

Supplementary Figure 2 Survival analysis of 13 prognosis-related m7GRGs.

Supplementary Figure 3 Immunohistochemical results of some m7G regulatory genes in adjacent normal and gastric cancer tissues. N represents normal tissue and T represents gastric cancer tissue.

Supplementary Figure 4
**(A)** lasso regression analysis based on 79 subtype-associated DEGs. Relationship between risk score and survival in GC patients of different age (**B**), gender (**C**) and tumor stage(**D**). (**E**)Relationship between m7GRG_score and clinicopathological factors, such as age, gender and tumor stage.

Supplementary Figure 5 The patients in stage I (A), stage II (B), stage III (C) and stage IV (D) were scored and grouped respectively, and then the survival, TMB and MSI analysis were performed in the high and low risk groups of each stage.

Supplementary Figure 6 Analysis of immune cell infiltration **(A)**, immune checkpoint expression(**B**), stromal score, immune score, ESTIMATE Score and tumor purity(**C**) between high- and low- risk groups. (**D**) Relationship between m7GRG_Score and TIDE scores.
