# Supplementary figures and images for: Identification of N7-methylguanosine related subtypes and construction of prognostic model in gastric cancer
Source: Front Immunol. 2022 Oct 10;13:984149. doi: 10.3389/fimmu.2022.984149 (PMC9589367; doi:10.3389/fimmu.2022.984149)

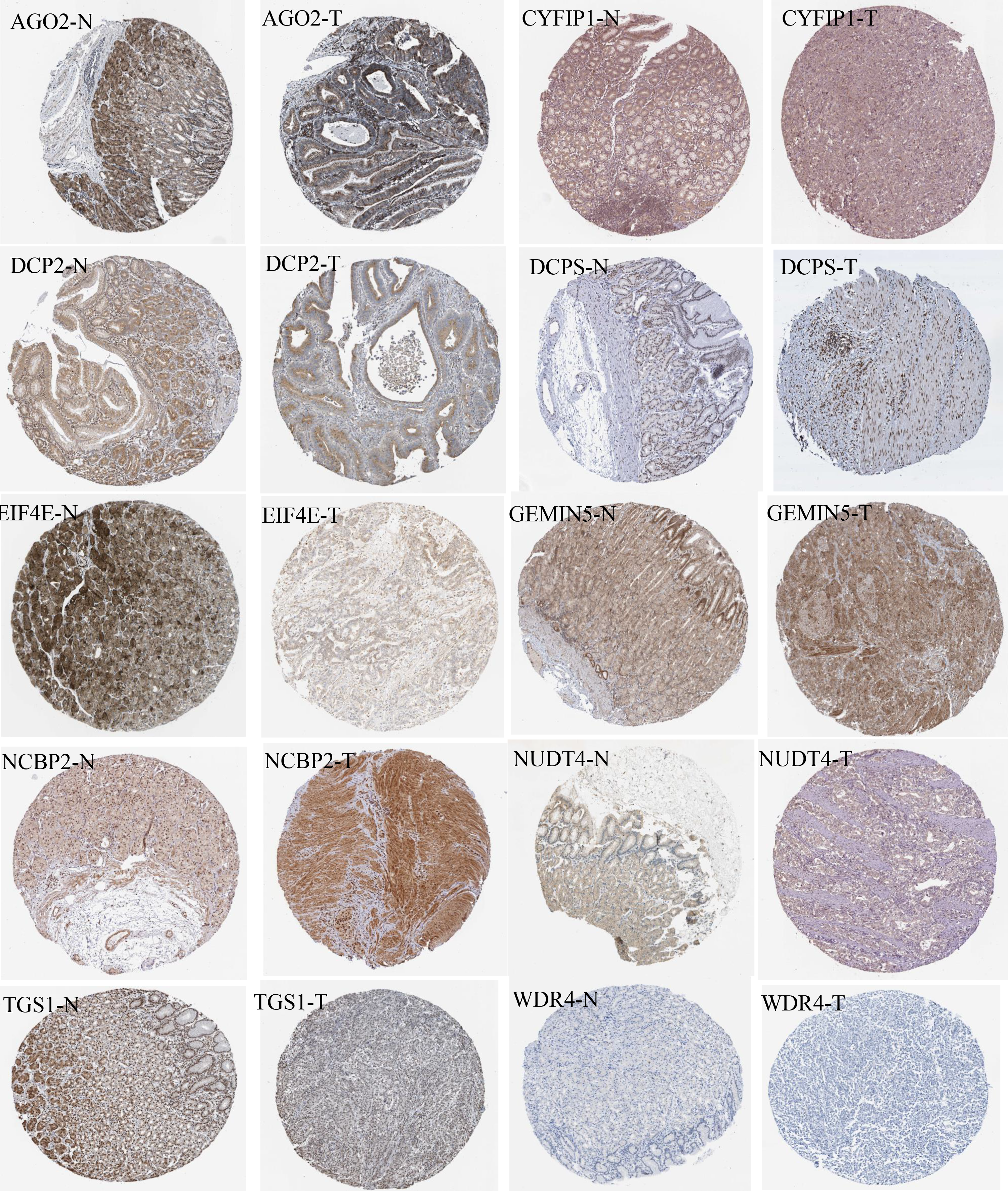

Supplement: Supplementary Figure 3 — Immunohistochemical results of some m7G regulatory genes in adjacent normal and gastric cancer tissues. N represents normal tissue and T represents gastric cancer tissue. [file Image_3.tif]

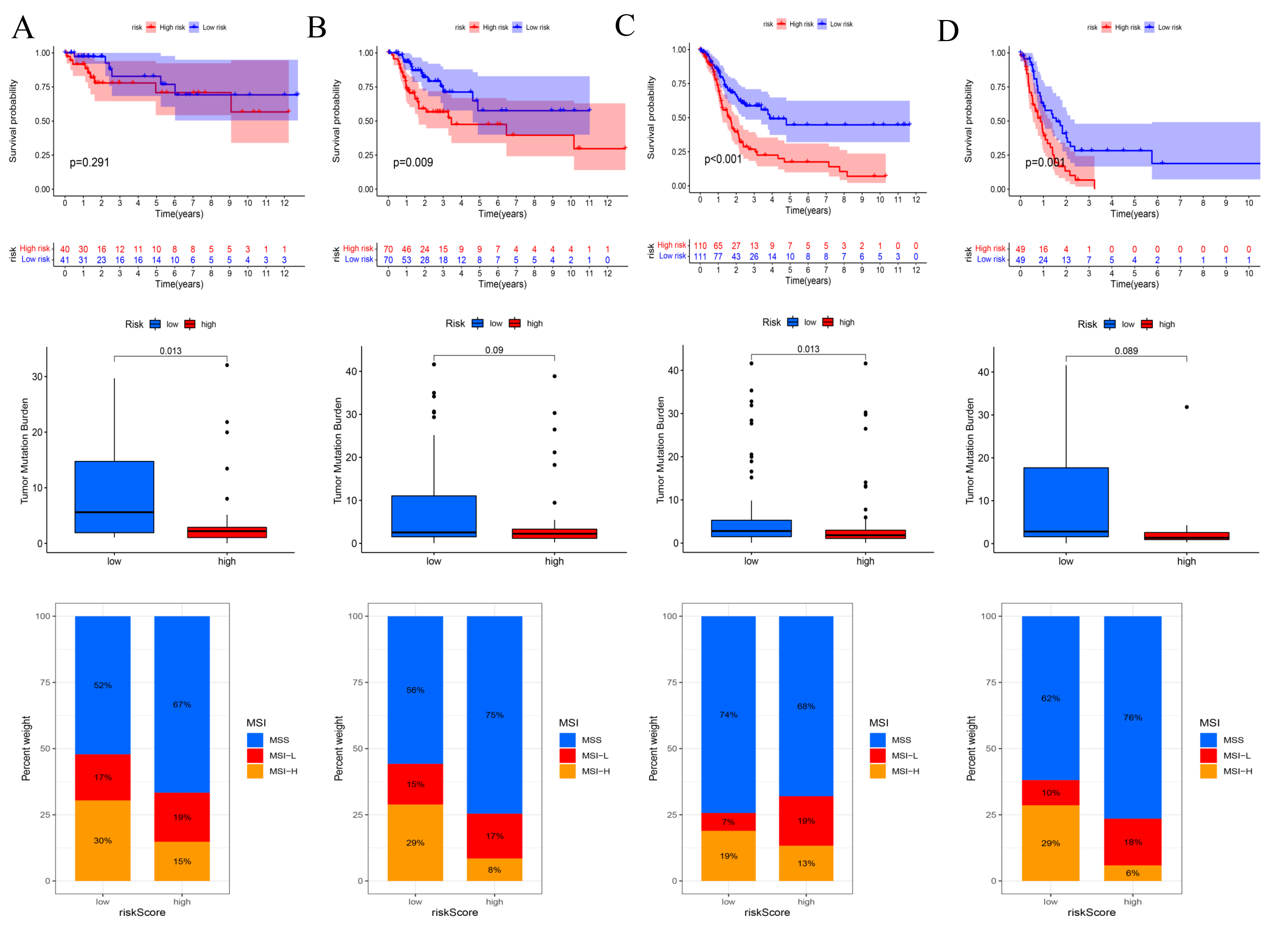

Supplement: Supplementary Figure 5 — The patients in stage I (A), stage II (B), stage III (C) and stage IV (D) were scored and grouped respectively, and then the survival, TMB and MSI analysis were performed in the high and low risk groups of each stage. [file Image_5.tif]
